# Epidemiology and clinical characteristics of hand foot, and mouth disease in a Shenzhen sentinel hospital from 2009 to 2011

**DOI:** 10.1186/1471-2334-13-539

**Published:** 2013-11-13

**Authors:** Yan-rong Wang, Lu-lu Sun, Wan-ling Xiao, Li-yun Chen, Xian-feng Wang, Dong-ming Pan

**Affiliations:** 1 Department of Pediatrics, the Affiliated Shenzhen Third Hospital, Guangdong Medical College, Shenzhen 518020, China; 2 Department of Prevention and Health Care, the Affiliated Shenzhen Third Hospital, Guangdong Medical College, Shenzhen 518020, China

**Keywords:** Hand, foot, and mouth disease, Epidemiology, Clinical characteristics

## Abstract

**Background:**

We investigated the epidemiological and clinical data of all hand, foot, and mouth disease (HFMD) cases in a sentinel hospital of Shenzhen, China from 2009 to 2011.

**Methods:**

HFMD cases diagnosed in our institution were assessed from 2009 to 2011. Both epidemiological and clinical features were analyzed retrospectively. All the fatal cases were reported.

**Results:**

A total of 12132 patients were diagnosed with HFMD, of which 2944 (24.3%) were hospitalized. Of the 2944 hospitalized patients, the highest proportion of diagnosed cases were admitted in May and July (989/2944, 33.6%). In 2009 all severe HFMD cases were diagnosed with enterovirus 71 (EV71). In 2010 and 2011, some of the severe HFMD were diagnosed with Coxsackievirus A16 (CA16). Incidence was highest in 0-4-year old children, with males being predominant. There were sporadic cases with HFMD the whole year except in February. All cases were cured in 2009. Six deaths were reported during 2010 and 2011.

**Conclusions:**

EV71 can cause severe complications and deaths in our region. HFMD is an important public health problem in Shenzhen in spite of stringent measures taken in preschool centers. A high degree of vigilance should be maintained over the disease situation.

## Background

Hand-foot-mouth disease (HFMD) is a common acute viral illness which is characterized by fever, oral ulcers, and vesicular exanthema on the hands, feet and buttocks. HFMD usually resolves spontaneously, but severe cases with complications such as encephalitis [1], pneumonia, myocarditis, brain-stem encephalitis and acute flaccid paralysis (AFP) have been reported in Asia recently [2-5], thus it has become a public health problem. Nationwide HFMD outbreaks have occurred in China since 2008 and HFMD has been made a nationally notifiable disease. Human enterovirus 71 (EV71) and coxsackievirus A16 (CA16) are the most common causes of HFMD and may cause potential life-threatening neurological and systemic manifestations. It is unfortunate that there is no effective vaccination or chemoprophylaxis for EV71 infection or HFMD. Favorable personal hygiene and isolating infected cases may be useful in public health prevention and control. We report a retrospective study of the epidemiology and clinical features of HFMD brought to our hospital for treatment from 2009 to 2011 to better guide public health action and practitioners.

## Methods

### Ethical consideration

This study was approved by the Ethics Committee of Affiliated Shenzhen Third Hospital, Guangdong Medical College, Shenzhen, China, on March 23, 2012. All the patient samples were taken as part of standard care.

### Clinical definitions

We reviewed the records of all cases of HFMD diagnosed in our hospital (Affiliated Shenzhen Third Hospital, Guangdong Medical College) by medical practitioners between January1st 2009 and December 31^st^ 2011. HFMD is characterized by oral vesicular exanthema/ulcers plus vesicular lesions on the hands, and/or feet, and/or buttocks. Meningoencephalitis [6] was indicated as cerebrospinal fluid (CSF) pleocytosis (white blood cell count, >5/mm^3^), with or without parenchymal lesions or substantial meningeal enhancement as identified by brain computed tomography or magnetic resonance imaging and/or by the presence of definite neurological dysfunction without CSF pleocytosis. Severe HFMD was defined as a patient with clinical features such as high fever, myoclonus, encephalitis, AFP, pulmonary edema, or heart failure [7,8].

### Laboratory notifications of enterovirus

EV-A71 and CV-A16 were routinely detected in our institution since 2009. Examinations for the viruses were carried out with stools, throat swab or cerebrospinal fluid collected from the patients.

A commercial licensed kit (Da An Gene Co. Ltd, lot no: Cox A16 YZB-0354-2009, EV-A71 YZB-0356-2009) was recommended by China CDC for detection of EV-A71 and CV-A16. The detection method is based on one-step real-time RT–PCR assay. The detection sensitivity of the kit is 1 × 10^3^ p.f.u/ml. A sample is considered positive for virus if reaction growth curves cross the threshold line within 35 · 1 cycles.

### Statistical analysis

Statistical analyses were performed using SPSS Software Version 15.0 (SPSS Chicago, IL). We used χ2 test for categorical data and Mann–Whitney U test for continuous non-parametric data. A Pvalue < 0.05 was considered statistically significant.

## Results

### Gender and age distribution of HFMD patients

A total of 12132 cases were reported during the period. Of 12132 HFMD cases, 7786 patients are boys and 4346 girls. There was a male predominance of HFMD cases, with a male-to-female ratio between 1.69:1 and 1.85:1 (Table 1). Although the ratio did not change significantly with time (P = 0.077), it suggested that boys have relatively higher chance to be infected by HFMD viruses than girls. 98.0% of the cases were younger than 10 years old. The proportion of 0 to 4-year-old children was largest, accounting for 83.3%, 88.6% and 91.3% of the reported cases in 2009, 2010 and 2011 respectively (Table 2). Children aged older than 5 years constituted only 16.7% of cases in 2009, and then this figure declined to 11.4% and 8.7% in 2010 and 2011 respectively (P = 0.000).

**Table 1:** Gender-Specific proportion of reported HFMD cases, 2009-2011

**Gender**	**2009**	**2010**	**2011**	**Total**
Male	1114(64.9)	2896(62.9)	3776(65.0)	7786(64.2)
Female	602(35.1)	1707(37.1)	2037(35.0)	4346(35.8)
Total	1716(100.0)	4603(100.0)	5813(100.0)	12132(100.0)

Figures in brackets refer to percentage of total cases in the corresponding year.

**Table 2:** Age distribution of HFMD cases between 2009 and 2011

**Group (y)**	**2009**	**2010**	**2011**	**total**
0– 4	1430(83.3)	4080(88.6)	5306(91.3)	10816(89.1)
5– 9	253(14.7)	420(9.1)	402(6.9)	1075(8.9)
10– 14	15(0.9)	49(1.1)	41(0.7)	105(0.9)
>14	18(1.1)	54(1.2)	64(1.1)	136(1.1)
Total	1716(100.0)	4603(100.0)	5813(100.0)	12132(100.0)

Figures in brackets refer to percentage of total cases in the corresponding year.

### Monthly distributions of reported HFMD cases

**Figure 1:**
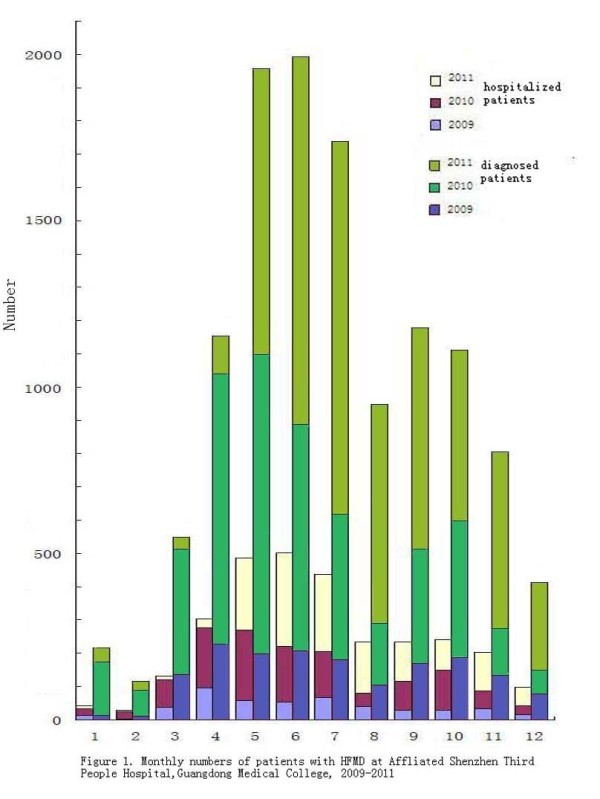
Monthly number of patients with HFMD at Affliated Shenzen Third People Hospital, Guangdong Medical College, 2009-2011.

Figure 1 shows that a seasonal peak appears between May and July every year. About half (46.9%) of HFMD occurred during this peak. A smaller peak was observed during September and October.

Only 24.3% (2944/12132) of patients needed to be hospitalized. The number of hospitalized cases in 2009 was 493. Then in 2010 and 2011, the number was 1155 and 1296 respectively. The hospitalized patients with HFMD were mainly admitted between May and July. Within that period, 503/2944 (17.1%) were admitted in June. May and July followed, with 486/2944 (16.5%) and 438/2944 (14.9%), respectively.

### Clinical characterization and laboratory diagnosis of hospitalized HFMD cases

1383/2944 (47.0%) were hospitalized due to poor feeding from high fever, mouth ulcers or vomiting. 190/2944 (6.5%) had severe clinical manifestations. 6/2944 (0.2%) of severe cases died.

The severe and fatal cases were most frequently seen in June, July and August. An equal number of fatalities occurred in June and July. The examination of throat swab and/or stool specimens from these 2944 patients revealed the presence of viral pathogens in 408 cases (408/2944, 13.9%).

Among the 408 patients, EV71 was predominant. The minority of the enteroviruses detected from the HFMD cases was CA16 (Table 3). EV71 were detected in all dead cases in our patients with HFMD.

**Table 3:** Viral distributions of hospitalized HFMD cases, 2009-2011

**Virus**	**2009**	**2010**	**2011**
EV71	57	148	175
CA16	0	8	20
Total	57	156	195

### Demographic features of infected people

The majority (79.0%) of cases occurred in those children who stayed at home. The percentage of patients in kindergartens and in schools was 17.9% and 2.9% respectively. 11 service workers and 1 physician were infected with HFMD in our patients.

### Mortality

Six HFMD-associated deaths were reported in May 2010 and 2011 when there was nationwide epidemic of HFMD. The first death involved a 4-month-old boy who got worse suddenly on the way to hospital and died on arrival at the Emergency Department. EV71 was isolated from the tracheal swab. The second death was a 1-year-old boy who jerked frequently at home and deteriorated suddenly at the Emergency Department. EV71 was isolated from the stool swab. The rest of 4 fatal cases were 2-year-old girls whose condition got worse rapidly with poor appetite and lethargy. They vomited and developed on the way to hospital. Clinically, they died of pulmonary edema. Throat swabs were detected positively with real-time PCR.

## Discussion

An EV71 outbreak caused 14 deaths in Linyi City of Shandong province and 23 deaths in Fuyang City of Anhui province in China since 2007. Dissimilar to Singapore’s report [9], EV71 is always the predominant pathogen in our patients with HFMD. 408 samples were positive for enterovirus (380 EV71 and 28 CA16). Although 8 and 20 cases infected with CA16 in 2010 and 2011 respectively, all the fatal patients were detected with EV71. Now that EV71 is known to be associated with fatal cases, the laboratory results should be monitored closely.

In this report, a total of 12132 HFMD cases were analyzed in Shenzhen, China between January 1, 2009 and December 31, 2011. Although the patients were reported in each month, about half of (46.7%) patients occurred in the period of May-July. It suggests that routine epidemiological surveillance and more preventive actions should be taken during the period of May-July of each year. A slight increase in number of HFMD cases was observed in September and October. The reason deserves consideration in future investigation. The report provided the evidence that patients younger than 5 years old represented more than 80% of the reported cases with HFMD. In contrast to the findings in other outbreaks [10,11], the proportion of HFMD was the highest in those children below 5 years of age who stayed at home which comprised 79.0% of the reported cases.

In Shenzhen, public health measures such as personal and environmental hygiene focus on kindergartens and preschools where highly susceptible children congregate. These institutions are required to report clusters of 5 or more cases of HFMD and recommended for voluntary closure to interrupt transmission of infection. Additionally, those infected children are kept at home until full recovery. Hence, the children in kindergartens only comprised 17.9% in our patients. Most of the children 1 year younger did not attend any day care schools and were principally taken care at home by family members, such as parents and relatives. The majority of these children never contact with people outside of their immediate family members. So it strongly suggested that those children infected the disease and enterovirus from their household members. Previous studies have indicated that intra-familial transmission occurs commonly [12,13]. Preventive public health actions should be taken to cover this target group. A male predominance of HFMD was observed in our study which is consistent with the precious reports [14]. The reason for the difference observed in gender incidence rates is still unknown and may attribute to the boys’ activity. We notice that boys are more susceptible than girls but the there are more female fatal cases than boys in our patients. Maybe parents focus more on sons for boys are favored and more valued than girls in Asian countries [15,16].

Despite the stringent measures taken to prevent transmission of HFMD in institutions where young children congregate, the number of cases with HFMD increases year by year from 2009 to 2011. However, due to the high degree of vigilance, institutional outbreaks were recognized early and rapidly brought under control with the majority of these outbreaks having an attack rate of less than 10%. Moreover, there had been a significant decline in the proportion of HFMD cases among preschool children aged between 0 and 4 years old over the years compared to older children aged between 5 and 9 years old. This suggests that control measures could have delayed the onset of infection.

Admissions to hospital requiring intravenous drip attribute to poor appetite due to mouth ulcers rather than neurological and cardiac complications. Of 493 cases admitted to our hospital during the 2009 epidemic, there were only 6 cases of aseptic meningitis. Similar to previous reports [17,18], EV71 was found to be more prevalent among the severely ill and hospitalized cases with HFMD.

It is possible that data collection may have been artificial and partial. In order to solve the problem, we have rationally designed the study and interpreted the results. Firstly, all patients were diagnosed in Shenzhen Third People’s Hospital which is the only institution in the city specializing in infectious diseases. So the majority of the patients that were suspected of HFMD and seen in other hospitals in the city were referred to the hospital. Secondly, each hospitalized patient was given a thorough medical examination, both throat swab and fecal samples were collected. Thirdly, to prevent negative results in the laboratory tests, each sample was detected with Real-Time-PCR assays instead of conventional reverse transcription PCR. To some extent, this study can represent the transmission and distribution of HFMD and viral infections in Shenzhen.

## Conclusions

EV71 can cause severe complications and deaths in our region. HFMD is an important public health problem in Shenzhen in spite of stringent measures taken in preschool centers. A high degree of vigilance should be maintained over the disease situation.

## Competing interests

The authors declare that they have no competing interests.

## Authors’ contributions

YW, XW and DP were involved in clinical diagnosis of patients. LS, WX and LC collected and chose the cases. YW drafted the manuscript. XW conceived the study, made the arrangements for obtaining cases from the hospitals, and finalized the manuscript. All authors read and approved the final manuscript.

## Pre-publication history

The pre-publication history for this paper can be accessed here:

http://www.biomedcentral.com/1471-2334/13/539/prepub
